# A Multicomponent Strategy to Improve HIV Pre-Exposure Prophylaxis in a Southern US Jail: Protocol for a Type 3 Hybrid Implementation-Effectiveness Trial

**DOI:** 10.2196/64813

**Published:** 2025-03-18

**Authors:** Ank E Nijhawan, Jana Kholy, Julia L Marcus, Timothy P Hogan, Robin T Higashi, Jacqueline Naeem, Laura Hansen, Brynn Torres, Barry-Lewis Harris, Song Zhang, Douglas Krakower

**Affiliations:** 1 Department of Internal Medicine Division of Infectious Diseases and Geographic Medicine University of Texas Southwestern Medical Center Dallas, TX United States; 2 University of Texas Southwestern Medical Center Dallas, TX United States; 3 Harvard Pilgrim Health Care Institute Boston, MA United States; 4 Center for Healthcare Organization and Implementation Research Veterans Affairs Bedford Healthcare System Bedford, MA United States; 5 Peter O’Donnell Jr School of Public Health University of Texas Southwestern Medical Center Dallas, TX United States; 6 Parkland Center for Clinical Innovation Dallas, TX United States; 7 Parkland Correctional Health Dallas County Jail Dallas, TX United States; 8 Division of Infectious Diseases Beth Israel Deaconess Medical Center Boston, MA United States

**Keywords:** pre-exposure prophylaxis, PrEP, HIV, HIV prevention, jail, incarceration, health disparity, electronic health records, EHR, southern United States

## Abstract

**Background:**

Pre-exposure prophylaxis (PrEP) is an effective approach for preventing HIV infection, but it is underutilized by populations who may benefit the most, including people living in the Southern United States and those involved in the criminal legal (CL) system. Improving the access and use of PrEP for these groups could decrease HIV-related health disparities. Beyond individual outcomes, HIV prevention for CL-involved people can have a significant public health impact on HIV incidence due to a high turnover between jails and the community.

**Objective:**

We will develop, implement, and evaluate a multicomponent PrEP implementation strategy for the Dallas County Jail (DCJ) to increase the initiation of this HIV-preventive intervention for CL-involved individuals.

**Methods:**

This is a type 3 hybrid implementation-effectiveness study that takes a combined approach by assessing the implementation of a strategy to identify candidates for PrEP at the DCJ and linking them to PrEP providers upon community re-entry while also gathering information about clinical outcomes. The approach is guided by the EPIS (exploration, preparation, implementation, sustainment) framework. Initial formative work (exploration) involves qualitative interviews of diverse key stakeholders to identify factors that may influence linkage to PrEP after jail release. These findings will undergo rapid qualitative analysis (preparation) to inform the adaptation of a multicomponent jail PrEP implementation strategy protocol. This approach, which will include an electronic health record (EHR) prediction model and integration of a PrEP patient navigator into the jail health team, will allow medical providers and the navigator at the DCJ to engage individuals most likely to benefit in shared decision-making about PrEP and navigate them to community PrEP care (implementation) in a process that begins before release from jail and ends with successful care linkage. Regular quantitative and qualitative evaluations of this approach will allow for ongoing stakeholder input, refinement of the implementation strategy, and maintenance of the program (sustainment).

**Results:**

Findings from 26 qualitative interviews (9 formerly incarcerated individuals, 9 county jail staff, and 8 employees of community organizations) have been obtained, analyzed, and mapped to an implementation strategy formalized in a jail PrEP protocol. An HIV risk prediction model based on EHR data to identify individuals most likely to benefit from PrEP has been developed and internally validated and is ready to be deployed. We anticipate the availability of preliminary study findings in 2026.

**Conclusions:**

This study will provide key insights into the feasibility and effectiveness of a PrEP implementation strategy among people at increased risk of HIV acquisition in an urban jail in Southern United States. This practical and scalable strategy can be used as a model for other urban jails to address HIV-related inequities.

**International Registered Report Identifier (IRRID):**

DERR1-10.2196/64813

## Introduction

It was estimated that there were 31,200 new HIV infections across the United States in 2021 [1], with a disproportionately high percentage of new diagnoses occurring in the South [2]. New HIV infections disproportionately affect men who have sex with men (MSM) and Black and Hispanic populations [3]. HIV prevention with pre-exposure prophylaxis (PrEP) can reduce the risk of HIV acquisition by 99% [4,5], but its public health impact depends on connecting populations at risk of HIV to PrEP care and continuing care over time [6-9]. Uptake of PrEP is increasing among White MSM on the east and west coasts but remains limited in minority groups in the South [10-12] where HIV incidence is the highest [13]. The PrEP-to-need ratio, defined as the number of PrEP users divided by new HIV diagnoses [14], is lower in the South than in any other region, particularly among racial and ethnic minorities, further highlighting inequitable access [15].

To decrease HIV incidence, there is a need to improve PrEP use in populations most heavily impacted by HIV. Launched in 2019, the federal *Ending the HIV Epidemic* (EHE) initiative provides an evidence-based framework for decreasing HIV incidence in the United States by 90% by 2030 [16]. The EHE initiative identifies that a critical component of epidemic control is to focus prevention efforts on geographic hotspots, including the 48 counties with the highest incidence, which are disproportionately in the Southern United States, and 7 states with major rural HIV epidemics. Active multisector partnerships among local public health departments, health care facilities, academic institutions, and community organizations are pivotal to achieving the EHE initiative’s goal [8,17].

Dallas County in Texas is one of the largest HIV hotspots in the South, with high rates of new HIV infections and major racial and ethnic disparities in HIV incidence and access to preventive services. In 2021, Dallas County had one of the highest rates of persons newly diagnosed with HIV at 38 per 100,000 population, compared to the national incidence of 11.5 per 100,000 population [18]. The rate of new HIV diagnoses was even higher for Black individuals in the county, at 72 per 100,000 population [19]. Despite the dense HIV epidemic in Dallas County, only 25% of the estimated number of people with indications for PrEP were prescribed it in 2023 [3]. Barriers include high rates of uninsurance and poverty [20,21], medical mistrust and stigma [22,23], low HIV risk perception [15], and high rates of incarceration [24].

About 1 in 7 people with HIV pass through US jails and prisons each year, making the justice system a potentially high-impact setting for HIV testing and prevention [25]. In Texas, the Department of Justice estimated in 2021 that 1.4% of the prison population is living with HIV, which is 4 to 5 times greater than the prevalence in the general US population [26]. Individuals in the criminal legal (CL) system, including those who are in jail, in prison, or under community supervision (ie, probation and parole), also remain at disproportionate risk for HIV infection due to higher rates of sexually transmitted infections (STIs) [27], condomless sex [28,29], transactional sex [30], shared injection drug use equipment [31], dissolution of primary intimate partnerships [32], and broader socioeconomic barriers to medical care (eg, unemployment and homelessness) [33]. Opt-out HIV testing in jails is feasible and can identify many new infections while avoiding the burden of requesting a test among patients [34]. Testing uptake when offered in a nonjudgmental opt-out manner is 80% to 95% [35-37] and should be followed by linking incarcerated individuals to appropriate HIV treatment or preventive care [38-42]. Yet, despite recommendations for the practice of opt-out HIV testing by the Centers for Disease Control and Prevention (CDC), only about a third of US jails offer opt-out HIV testing [43].

Given that most individuals spend less than a month in jail and often cycle multiple times between incarceration and the community [44], there is a high potential public health impact of HIV prevention strategies in jails. Barriers to PrEP implementation in jails exist at several ecological levels and include limited knowledge of PrEP among those likely to benefit and their providers [45,46], lower perceived than actual risk of HIV [47,48], and distrust of institutions [49]. The timing of HIV prevention interventions is also significant, with the transition to living in the community being a particularly pivotal period when HIV transmission–risk behavior is increased and other personal needs take priority during community re-entry [50,51]. For these reasons, effective PrEP implementation strategies in jails may require multiple components to be successful [52-54]. For example, interventions at the individual level have included supporting HIV prevention after incarceration through a mobile app with customized wellness goals and incentives [55]. Building on evidence from CL-involved people with HIV transitioning to the community, peer education and patient navigation are being explored as approaches to improving HIV prevention [56-59]. At the dyadic level, patient-provider shared decision-making around PrEP, which has been used in the community, is also being applied in correctional settings [60-63]. The objective of our study is to assess specific needs and recommended strategies and use these findings to develop, implement, and evaluate a multicomponent PrEP implementation strategy for the Dallas County Jail (DCJ), which is the 8th largest jail in the nation and located in an HIV hotspot [64].

## Methods

### Study Setting

The DCJ is the main jail facility for Dallas County, with 51,000 incarcerated individuals per year (86% men, 48% Black, and 30% Hispanic) and approximately 275 individuals entering and leaving the jail daily. The mean length of stay is 45 days, and 11% of people transfer to prison [65]. Parkland Health, a teaching hospital for UT Southwestern Medical Center, provides health care services at the jail for approximately 6200 patients monthly. Health care for individuals experiencing incarceration is paid for by Dallas County.

Of over 12,000 HIV/STI tests performed each year at the DCJ, there are approximately 50 to 100 new HIV diagnoses (personal communication by Dr Barry-Lewis Harris, April 2024) and high STI positivity (5% syphilis, 5% gonorrhea, and 11% chlamydia) [35,66], which is a recommended indication for PrEP use. The approach to HIV testing at the DCJ is a combination of opt-in and opt-out. All persons entering the jail complete a nursing intake questionnaire to review medical history, receive the option for HIV/STI testing (opt-in), and, for men, indicate if they want to be in separate housing for MSM as a safety precaution. All individuals who undergo blood draws are offered opt-out HIV testing. Individuals with negative HIV test results are not routinely counseled about the results, and few, if any, receive information about PrEP. Currently, new initiation of PrEP is not permitted at the DCJ, and thus, the study will focus on linkage to PrEP after release.

### Study Design

**Figure 1:**
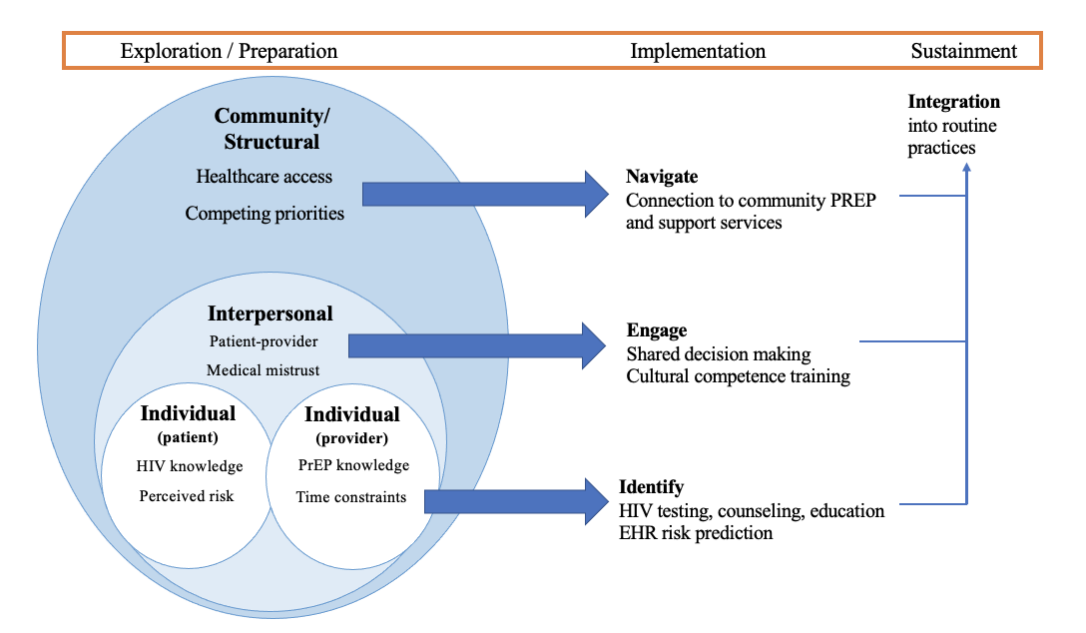
Conceptual socioecological model of pre-exposure prophylaxis (PrEP) implementation for criminal legal–involved people. EHR: electronic health record.

This is a type 3 hybrid implementation-effectiveness study, which takes a combined approach in assessing implementation while also gathering information about clinical outcomes associated with implementation [67]. We will track established implementation outcomes (eg, feasibility, acceptability, and penetration), effectiveness (eg, attending PrEP visits and new HIV/STI diagnoses), and sustainability, including integration into routine processes at collaborating agencies and duration of any positive effects in care processes and outcomes. We will use the EPIS (exploration, preparation, implementation, sustainment) framework [53,54] to guide our efforts to identify and address performance gaps in DCJ PrEP care. Although originally developed for public service sectors, the EPIS framework has since been widely applied in health care, including in jails [54]. As shown across the top portion of Figure 1, our key study concepts and activities reflect EPIS framework components.

### Study Aims

#### Aim 1: Exploration

Using the socioecological model as a conceptual model [68], we will identify and describe specific individual, interpersonal, and community/structural factors that influence PrEP implementation through qualitative interviews and focus groups with key stakeholders in the jail and Dallas community (Figure 1).

##### Participant Characteristics and Recruitment

Participants will be individuals aged 18 years or older who are medical providers or administrators in the DCJ or in the surrounding community, people formerly incarcerated in the DCJ, representatives of community-based organizations serving those affected by HIV or with criminal justice involvement, and members of the public health department. Of note, due to restrictions on recording individuals at the DCJ and due to the benefit of addressing the postincarceration transition, the individuals we recruit will be formerly incarcerated rather than currently incarcerated. Formerly incarcerated individuals will be referred by their health care providers in community clinics or can self-refer in response to Institutional Review Board (IRB)-approved flyers posted in community-based service organizations. Health care professionals will be recruited at routine staff meetings and via email invitations. Administrators will be contacted about participation via email or personal contact from members of the study team.

##### Data Collection

We will conduct 20 to 30 semistructured interviews, which, based on our previous work, should be sufficient to reach saturation (ie, the point at which no new information is being uncovered through additional interviews). Individual interviews are preferred for formerly incarcerated individuals, jail staff, and members of community organizations due to the sensitive nature of questions and the convenience of scheduling.

Separately, we will also conduct a focus group with 10 to 12 members of the HIV/AIDS Re-Entry Coalition (HARC) in Dallas County during one of their quarterly teleconferenced meetings. The HARC is a group comprised of individuals from diverse community organizations who regularly collaborate to enhance the delivery of health and supportive services to individuals who have been recently released from incarceration. Feedback is especially valuable from these group discussions given the breadth and length of experience of the members. Lastly, we will conduct 2 focus groups during a summit event in which we will invite PrEP-providing organizations to compare their experiences and challenges in providing PrEP and related services amid the dynamic funding landscape and to assess opportunities for collaboration.

Semistructured interview and focus group guides will be tailored to each target group, and topics will reflect the range of factors relevant to PrEP implementation across different socioecological levels, as shown in Figure 1. All participants will also be asked to provide feedback on how to adapt and optimize the components of our implementation strategy for local populations, settings, resources, and norms. All interviews and focus groups will be audio recorded and conducted by research staff experienced in qualitative methods.

##### Data Analysis

We will conduct both a rapid qualitative analysis during data collection and a systematic thematic analysis when all data have been collected. The purpose of the rapid analysis is to promote consistent data capture, facilitate communication among the members of the research team about findings, and monitor progress toward saturation [69]. The thematic analysis will systematically categorize patterns in findings across interviews within a group (eg, patients) and across groups (eg, patients, jail providers, and health care leaders), using iterative thematic analysis in accordance with best practice techniques [70,71]. All data will be managed in NVivo 12.0 (QSR International). The study’s principal investigators in conjunction with a study co-investigator who is an established qualitative methodologist will jointly develop a thematic codebook based on a review of the rapid analysis data and 20% of the interview fieldnotes, with codes mapped to the socioecological model presented as part of Figure 1. Trained research staff will double-code all interview transcripts, resolving discrepancies through discussion and conferring with the qualitative co-investigator as needed. A synthesis of the major findings will be presented to stakeholder groups to confirm appropriateness [72].

#### Aim 2: Preparation

In aim 2, our qualitative findings from aim 1 will inform the refinement of a multicomponent PrEP implementation strategy for CL-involved individuals. We will adapt and hone approaches to identify incarcerated people at increased risk for HIV using electronic health record (EHR) clinical decision support tools and patient-reported data to prompt providers to conduct HIV/STI tests and discuss PrEP with those most likely to benefit; engage CL-involved individuals in shared decision-making about PrEP using adaptations of previously developed patient decision aids; and navigate these individuals to existing PrEP services in the Dallas area after release.

##### Datasets to Identify Individuals at Increased Risk for HIV

The Parkland Center for Clinical Innovation (PCCI) has developed and validated an automated matching algorithm to extract data from the DCJ EHR (PEARL) and match it to data from Parkland’s EHR (Epic) from 2010 to the present. Records will be matched based on multiple variables, including patient name, preferred name, date of birth, gender, sex assigned at birth, race or ethnicity, and social security number as available. We will exclude any individuals younger than 18 years. Individuals may have multiple encounters in both the jail and Parkland EHRs due to repeat jail incarcerations or medical visits, and these data will be incorporated as summary variables (eg, number of prior episodes of gonorrhea) with statistical adjustments for repeated measures in an individual. This combined dataset will provide rich data for identifying PrEP candidates and allow us to develop an HIV prediction model. This component of the study will involve the collection of existing data and the development of tools and protocols, and therefore, a waiver of consent will be obtained. Furthermore, matching of records using the identifiers above will be performed behind the firewall of the PCCI, and all datasets will be deidentified prior to sharing with the rest of the research team.

##### Validation of the HIV Risk Prediction Model

We will use our prior models for predicting incident HIV [73,74] to identify candidate variables for our HIV prediction model. Subsequently, the use of temporal validation will assess how well our model will perform prospectively, dividing our dataset into a derivation dataset (2015-2021) and a validation dataset (2022-present). We will train our model to predict incident HIV diagnoses, defined as positive HIV screening and confirmatory tests or new HIV diagnosis codes without prior evidence of HIV in the EHR [73-75]. We will have ample data on new HIV diagnoses to train our models, as Parkland has a robust HIV testing program and diagnoses more new cases of HIV than any other entity in Dallas [76]. Jail EHR data may be more limited in scope because the jail generally provides short-term episodic care. However, given high recidivism rates [77], many incarcerated individuals will have multiple data points in the jail EHR to inform HIV risk prediction, including data on prior HIV/STI testing and substance use.

We will compare the performance of parsimonious versus full models in the validation dataset based on their ability to discriminate between patients with and those without incident HIV using the C-statistic or area under the receiver operator characteristic curve and the Hosmer-Lemeshow goodness of fit test. Additionally, we will use multiple complementary approaches for estimating HIV risk to capture the greatest number of individuals with indications for PrEP. This will include anyone who identifies as transgender, requests MSM housing, has had a recent STI, or opts-in for HIV/STI testing, in addition to those flagged by our EHR model. We will compare EHR models against these criteria to measure the added value of each approach (eg, number of individuals identified by our model as likely to benefit from PrEP who are missed by patient-reported data and vice versa).

##### Protocol Development

In conjunction with our risk prediction model, the findings from our aim 1 stakeholder interviews will directly inform our protocol for identifying persons who are at increased risk for HIV and likely to benefit from PrEP. This will include elements, such as which practitioners receive EHR alerts about potential PrEP candidates and the content of staff trainings for collecting and acting on patient-reported data and EHR alerts. We will select a final process for identifying candidates for PrEP discussions based on sensitivity (ie, missing as few persons at increased risk as possible), feasibility, and efficiency, while taking into account priorities identified by stakeholders in aim 1.

A key consideration for sexual health discussions in the jail setting is privacy. Feedback in aim 1 will inform how and where these discussions will take place. Providers may also desire additional training in assessing sexual risk, as knowledge of PrEP among correctional nursing staff and clinical providers is vital for successful implementation [78]. Furthermore, the approach to incarcerated individuals about sensitive topics, such as sexual health and substance use, will be informed by best practices, including having these screenings conducted one-on-one by medical staff and partnering with community-based organizations [79]. Any paper materials given to patients will be adapted to the American Medical Association’s recommended 6th grade reading level and worded to avoid disclosing the health status.

##### Navigation for Individuals Likely to Benefit From PrEP

Individuals who are identified as being at increased risk for HIV and who test negative for HIV will be considered PrEP candidates. We will develop a PrEP decision-support tool to be used with the diverse populations present in the jail, informed by stakeholders’ preferences from aim 1. This will include presenting information on sexual health and HIV prevention for multiple populations (eg, transgender people and cisgender women), local details on HIV epidemiology, and ways to afford and access PrEP. Individuals identified as PrEP candidates will meet with medical providers or navigators (depending on stakeholders’ preferences) to review HIV prevention options and our patient decision-support tool. Patients will be informed that they will not be charged for these visits, as for all care related to communicable diseases in the DCJ. At this visit, the provider or navigator will assess the patient’s knowledge of and interest in PrEP. Together with the patient, they will work through the decision-support tool. If the patient plans to pursue PrEP care after incarceration, the navigator will initiate the navigation protocol.

##### PrEP Care Navigation and Linkage Following Release

Those interested in PrEP will receive intensive navigation that includes both in-person and electronic referrals. Similar to existing navigation protocols (eg, for hepatitis C [80]), the navigator will initiate this process while the individual is still in jail. The navigator will obtain detailed patient contact information, identify and address the patient’s health priorities and potential barriers to care, and create a personalized step-by-step process for accessing PrEP services after release. Patients will also be able to contact the navigator after jail release directly by phone or SMS text messaging.

Multiple PrEP providers in the Dallas community employ peer outreach workers who accompany patients to their PrEP visits. We will work with these providers to develop a protocol for seamless hand-off from the jail-based navigator to the community-based peer. For electronic referrals, we will integrate stakeholders’ preferences for details regarding the transfer of information to community PrEP sites, including what information to communicate, how to communicate, and the timing of communication. Findings from our aim 1 qualitative data will help us pinpoint solutions to barriers or competing priorities to PrEP that patients identify. In some cases, we anticipate that multiple referrals will be necessary for patients to successfully access PrEP.

#### Aim 3: Implementation and Sustainment

In aim 3, we will deploy our multicomponent strategy at the DCJ and assess its impact on implementation and clinical outcomes for PrEP, as specified in Table 1.

**Table 1:** Quantitative implementation outcomes, metrics, timing, and data source.

Hybrid type 3 domain and outcome		Metrics (among patients with high risk)	Timing (month range)	Data source^a^
**Implementation**				
	Feasibility	HIV/STI^b^ testing offered	1-24	EHR^c^, navigator
	Feasibility	PrEP^d^ discussions offered	1-24	EHR, navigator
	Feasibility	Shared decision-making tool offered	1-24	EHR, navigator
	Feasibility	PrEP navigation visits offered	1-24	EHR, navigator
	Feasibility	PrEP referrals offered	1-24	Navigator
	Penetration	Providers discuss/refer for PrEP	1-24	EHR, navigator
	Acceptability	HIV/STI testing completed	1-24	EHR
	Acceptability	PrEP discussions completed	1-24	EHR, navigator
	Acceptability	Shared decision-making tool used	1-24	EHR, navigator
	Acceptability	PrEP navigation visits completed	1-24	EHR, navigator
	Acceptability	PrEP referral completed	1-24	Navigator
**Effectiveness**				
	PrEP	PrEP visits in the community	1-24	Navigator
	PrEP	PrEP prescription	1-24	Navigator
	PrEP	PrEP use at 3 months	1-24	Navigator
	HIV/STI	New HIV/STI diagnoses	1-24	EHR
**Sustainment**				
	All	Reassess all study outcomes	25-30	All

^a^Qualitative assessments (3 times per year) complement quantitative metrics for all outcomes.

^b^STI: sexually transmitted infection.

^c^EHR: electronic health record.

^d^PrEP: pre-exposure prophylaxis.

##### Training of DCJ Staff

The project principal investigators will conduct a 2-session interactive group training for frontline DCJ practitioners to introduce our PrEP implementation strategy. Session 1 will be adapted from trainings on culturally competent sexual health care, HIV, and PrEP [81], including clinical indications for PrEP and barriers and facilitators to its adoption in the jail and local community. Session 2 will introduce the rationale and practical aspects of using patient-reported and EHR data to identify PrEP candidates, as our prior qualitative work suggests that providers are more likely to trust, adopt, and use HIV risk assessment tools if they understand how they work [82]. The session will also review how providers and navigators can engage patients in the shared decision-making process and will include demos of PrEP decision-support tools. Lastly, session 2 will cover PrEP navigation processes, including PrEP resources in Dallas County, and navigation protocols. Postsession evaluations will be conducted for rating quality and usefulness.

##### Steps for Strategy Implementation

**Figure 2:**
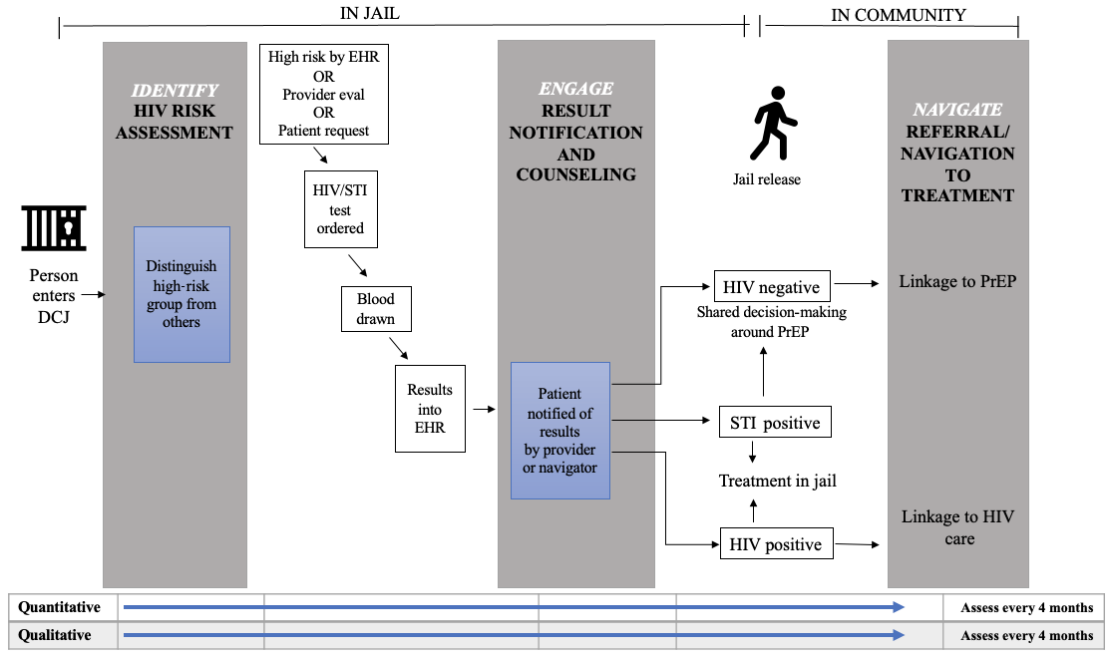
Intervention steps in the jail-to-community continuum. DCJ: Dallas County Jail; EHR: electronic health record; PrEP: pre-exposure prophylaxis; STI: sexually transmitted infection.

Our strategy will include the following detailed steps that can be visualized as part of the continuum (Figure 2): (1) identify, (2) engage, and (3) navigate.

In the “identify” step, when individuals enter the jail, a jail EHR record will be created (or an existing record will be opened for those with prior DCJ incarceration). Our automated algorithm will match the jail EHR to the Parkland EHR and generate an individual HIV risk score. For people with predicted HIV risk above a prespecified threshold that maximizes sensitivity without causing alert fatigue among providers, an automated prompt will appear in the jail EHR for providers to discuss PrEP and order HIV/STI testing. As illustrated in Figure 2, providers will also be prompted to order HIV/STI tests for any patients who report risk factors at intake or during a clinical assessment (eg, transgender identity and requests to be housed with MSM) or who request testing directly. Prompts will persist until orders are completed as a “nudge,” a behavioral economic intervention to maximize ordering [83], and results are entered into the EHR.

In the “engage” step, positive HIV tests will prompt referrals to the jail HIV clinic for rapid initiation of antiretroviral therapy according to the current practice, and STIs will be treated based on CDC guidelines [84]. Persons with negative HIV tests will meet one-on-one with a provider or PrEP navigator and receive counseling informed by our PrEP decision-support tool. The navigator we hire will be experienced in working with CL-involved people. Counseling will be person-centered and culturally tailored, with positive sexual health framing.

In the “navigate” step, for patients interested in PrEP, the navigator will review a list of PrEP clinic locations and assist with selecting a provider. Once an individual is released from the DCJ, the navigator will facilitate PrEP linkage by contacting that individual (by phone or SMS text messaging) and providing personalized care navigation. A hotline will be available for released individuals to call for assistance with PrEP referrals. The navigator will continue to follow referred patients for 3 months after any PrEP initiations, recording these contacts and clinical outcomes in REDCap, a secure online data capture system [85].

For this aim of the study, the patient navigator will obtain verbal consent from patients to proceed with a discussion on sexual health and to contact them after release from jail. The navigator will use a script to inform patients about the goals of the project, including the metrics to be collected, and reinforce that the meeting with the navigator is voluntary and will not affect the legal status of the patients.

We will track quantitative and qualitative metrics related to our implementation strategy during a 24-month period of iterative assessment and refinement. Our study metrics map directly to the EPIS framework at key steps in the PrEP care continuum (Figure 2). As shown in Table 1, we will assess quantitative effects, including the number of patients who attend a community PrEP visit, initiate PrEP, and persist with PrEP use for ≥3 months, which is a robust HIV prevention outcome [7]. We will track the rates of new and previously known HIV diagnoses and STIs in the DCJ through EHR chart review.

##### Feasibility

Feasibility refers to the extent to which a practice change can be successfully used or carried out within a specific setting [86]. We define feasibility as >65% of eligible patients being offered HIV/STI testing and one or more PrEP discussions, our patient PrEP decision-support tool, a PrEP navigation visit, or a PrEP referral in jail.

##### Penetration

Penetration refers to the extent to which a practice is integrated within a specific setting [86]. For this metric, we will track the proportion of providers who discuss PrEP or refer to a navigator for at least one eligible patient.

##### Acceptability

Acceptability refers to the perception among setting stakeholders that a practice is agreeable, palatable, or satisfactory [86]. We will calculate the proportions of patients who accept offers for one or more HIV testing, STI testing, PrEP discussion, navigator contact, or PrEP referral, with acceptance of >80% of test offers and >65% of discussion offers considered acceptable based on prior studies [35,87-89]. We will evaluate differences in feasibility and acceptability among patients by age, sex, gender identity, and race or ethnicity to identify imbalances in the offering and uptake of testing and referrals.

##### Effectiveness Regarding PrEP Outcomes

Additionally, on a monthly basis, we will measure the proportion of individuals who test negative for HIV, the proportion of this subgroup who receive notifications of their HIV test results, and the proportion of all those tested who are candidates for PrEP based on US Public Health Service practice guidelines for PrEP [90].

Among these PrEP candidates, we will assess how many are released to the community and how many are successfully contacted by the jail navigator after release. We will track the proportion of candidates attending ≥1 PrEP visit after jail release, the proportion prescribed PrEP, and the proportion using PrEP at 3 months after initiation (using direct patient assessments by the navigator). We will complete a run chart, which we will adapt from quality improvement methodologies [91], to evaluate trends in the PrEP care cascade [92] on a monthly basis during the 24-month intervention period and the additional 6-month sustainment period.

##### Effectiveness Regarding New HIV/STI Diagnoses

We will also track STIs and new and previously known HIV diagnoses in the jail among priority groups and the general jail population. STI positivity will be calculated as positive tests per the total number of tests completed. New HIV diagnosis rates will be calculated as confirmed positive test results in persons without a prior diagnosis of HIV per the total number of tests completed within a given time period. We will use an interrupted time series analysis using monthly data points to compare rates during the 24 months before and 24 months after our intervention is implemented. Our primary analysis will involve a comparison of the longitudinal rates before and after the intervention, and a mixed effects model for interrupted time series will be used as a secondary analysis. We will conduct linear regression analyses to determine if there is a significant difference in new HIV diagnosis rates before and after the intervention.

##### Sustainment

Sustainability refers to the extent to which a new practice is maintained or institutionalized within a setting’s ongoing operations [86]. We will assess how well the strategy components are maintained as part of existing jail processes for 6 months after the formal 24-month study period, including tracking retention in PrEP care for up to 6 months (through navigator follow-up). Our research team includes dissemination consultants, who are medical providers working in jails in Austin, TX; Chicago, IL; and Boston, MA, and who will offer input on refining our strategy components for ongoing use at the DCJ and the potential transfer and integration into other jail settings.

For PrEP linkage, current rates of linkage are essentially zero. Thus, in lieu of a pre/post comparison, we will report the proportion of patients linked to PrEP care during the implementation period with 95% CIs. Power analysis is performed for the interrupted time series analysis of monthly HIV positivity rates, assessing the difference in the mean between the 24 months before and after implementation. Over the preimplementation period, an anticipated 250 tests will be performed monthly with 3 new HIV diagnoses per month. In the postimplementation period, of 500 HIV tests per month, an increase to 9 new diagnoses per month is anticipated due to the focus on individuals identified by the prediction model as being at increased HIV risk. Furthermore, we assume a correlation of 0.2 among monthly observations. Based on a mixed effects Poisson regression model, we can detect the difference in the mean HIV-positive rate between the 2 periods with >80% power at a 2-sided type I error of 0.05. We will also perform subgroup analyses, comparing testing rates by sex, age group, and race or ethnicity.

##### Evaluation of Qualitative Effects

We will also evaluate the qualitative effects of our implementation strategy through structured observations and discussions at the DCJ to assess changes (positive or negative) in knowledge and opinions of PrEP, workflow, and cross-agency collaboration in line with Dallas County’s goal of a coordinated HIV response [93]. Observations will be conducted by individuals with postgraduate training in qualitative methods who have undergone additional training with the DCJ and Parkland Health and who are approved to conduct observations under the protocol approved by the IRB at UT Southwestern Medical Center. Observations will be focused on specific interactions pertinent to the research questions (eg, between the navigator and person soon to be released). Qualitative data on effectiveness and implementation outcomes will be rapidly analyzed in real-time using structured analysis of field notes from observations and discussions. The resulting iterative findings will be presented to a study advisory panel every 4 months to assess evolving attitudes and practices surrounding HIV testing and PrEP (ie, acceptability), gather perceptions of feasibility and penetration of the intervention, and solicit feedback about the ways to improve outcomes.

#### Ethical Considerations

Each aim of the study has undergone a separate review of procedures and protocols by the UT Southwestern Medical Center IRB, with details of human subject protection outlined in the protocol by aim. This IRB has a prisoner representative, and all IRB-approved protocols were reviewed by Parkland Health and approved by the DCJ correctional health leadership before beginning study activities. To date, the IRB has approved the protocols for aim 1 (STU 2021-0763) and aim 2 (STU 2021-1172). In addition, the Office of Human Research Protection has reviewed and approved the inclusion of prisoners in human subject research for the overall study protocol in 2022.

For all participants in focus groups and individual interviews in aim 1, the research team has obtained verbal consent. To ensure informed consent, researchers also provided information about the nature and purpose of the study, confidentiality and privacy, HIPAA (Health Insurance Portability and Accountability Act) regulations, potential risks, discomforts and benefits of participation, and the option not to participate or start then stop participating in the interview at any time. In addition, stakeholders were encouraged to contact the study team at any time with questions or concerns they may have about the study. Eligible participants (ie, individuals not employed by UT Southwestern or Parkland Health according to institutional policy) were paid US $25 as compensation for their time for each interview or focus group attended.

Regarding EHR usage for aim 2, all study data were collected from existing EHRs, including medical intake forms at the DCJ and EHRs from Parkland Health and Hospital Systems, to identify potentially eligible subjects for the research. Records were matched based on multiple variables, including patient name, preferred name, date of birth, gender, biological sex, race or ethnicity, and social security number, as available. The medical record data include data collected as part of the medical intake process at the jail and routine clinical care at the jail and Parkland, including demographics, information from clinical encounters, prescription information, laboratory tests and results, and billing information. For those who did not receive medical care or treatment at Parkland Health, only data from the jail EHR were used. There was no additional direct collection of specimens, records, or data from subjects, and the IRB approval allowed secondary analysis without additional consent to identify research subjects and build the prediction model.

Data are not deidentified, as individuals meeting the basic eligibility criteria will be contacted by the researchers for recruitment into the study. No additional research procedures or continued access to identifiable private information will occur until after subjects have given valid consent to participate in the research study. The risk of this activity is minimal to the subjects because the information collected will be limited only to information allowing researchers to determine eligibility for the study and contact information. Only the minimum information necessary to determine eligibility will be recorded. Researchers have been granted access to the medical record data by the institutions and will protect the data they use and record for this activity according to institutional and HIPAA standards for protecting privacy and maintaining confidentiality. If the subjects identified decline participation in the research, the data collected will not become part of the research data. If subjects agree to participate in the research, the identifiable data collected will become part of the subjects’ research records and will be stored according to the research confidentiality plan. Because of the nature of the detailed inclusion criteria, it is not feasible for researchers to advertise and screen for eligible subjects. The Office of Human Research Protection highlights this as an acceptable activity to waive informed consent to permit investigators to obtain and record identifiable private information for the purposes of identifying potential subjects [94].

## Results

The study was funded by the National Institute of Mental Health in April 2022. Progress has been made on various aims to date. Qualitative interviews have been completed with 26 individuals, including 9 formerly incarcerated individuals, 9 county jail staff, and 9 employees of community organizations (1 individual was both a formerly incarcerated individual and an employee of a community organization). A focus group with 10 additional individuals has also been conducted. All interviews have been transcribed, coded, and analyzed, and a manuscript highlighting the key themes is in progress.

In addition, summaries of field notes for each interview have been organized into key barriers; facilitators; suggested approaches to overcome barriers; resources needed to overcome barriers; and specific strategies to improve risk assessment, HIV testing, sexual health or PrEP education during incarceration, and postrelease linkage to PrEP. Findings from these interviews have been applied to formulate a jail PrEP implementation protocol using rapid qualitative analysis.

An HIV risk prediction model based on jail EHR data to identify individuals most likely to benefit from PrEP has been developed using records from 33,458 individuals who have undergone HIV testing in jail. This model has been optimized using machine learning, has been internally validated, and is ready to be deployed. Future exercises will include integration with health system EHR data from Parkland Hospital and comparisons to parsimonious models, which will guide the iterative development of the model. We anticipate that preliminary study findings on the implementation and effectiveness of the jail PrEP implementation strategy will be available in 2026. 

## Discussion

### Expected Results

We anticipate that the implementation of the multicomponent strategy will promote robust linkage to PrEP care for many individuals at risk for HIV, which currently does not occur at the DCJ. Moreover, we expect to identify a higher number of new HIV infections and STIs than in the 2 years prior to implementation. Our final product will be a well-integrated sustainable multicomponent PrEP implementation strategy that starts upon jail entry and ends with linkage to community-based HIV prevention, which can address major HIV-related inequities for CL-involved populations.

### Potential Limitations and Alternative Approaches

In the process of refining the implementation strategy in our second aim, EHR models may be hampered by limited or missing data. We will minimize the missingness of EHR data by combining jail and Parkland data and extending this dataset over a 10-year timespan. We will explore the use of the missingness of EHR variables (which may be a proxy for not being engaged in primary care) as indicators to understand the effect on model performance. If model performance is suboptimal, we will rely primarily on patient-reported data to identify HIV risk. For EHR alerts, we will proactively address potential alert fatigue [95] by employing rigorous user-centered design principles [96]. We will also train providers on how to respond to alerts about HIV risk, as providers may be unaccustomed to these.

Delays in the generation of electronic HIV risk scores could result in the identification of individuals likely to benefit from PrEP after their release from jail. If this occurs, we expect to identify many of these individuals using patient-reported data. However, the model we have developed will generate scores on day 3 of each person’s incarceration, which will mitigate delays. We will also generate future EHR prompts for HIV/STI testing and PrEP if the patient returns to jail, and the navigator will contact the patient to encourage HIV testing and PrEP evaluations with community providers. Though patients may decline visits to discuss PrEP, we will maximize patient interest by training staff to present PrEP in a positive, discreet, and patient-centered manner and will offer our decision-support tool for patients to review independently. Since barriers, such as medical mistrust, transportation, and anticipated stigma, may limit patients’ access to PrEP, we will hire and train a navigator who is able to build rapport with diverse populations, use principles of trauma-informed care [97-99], and teach at many education levels. Additionally, we will access existing resources, such as care coordination, offered by community partners.

### Study Impact

Despite these possible limitations, this project will generate a valuable approach to prevent HIV in the disproportionately burdened population of CL-involved individuals in the Southern United States. As these individuals often have fragmented health care and may not be reached with approaches targeting primary care or STI clinics [100], our multicomponent implementation strategy has the potential to efficiently reach many persons likely to benefit from PrEP in a nontraditional but well-defined setting with rapid turnover to the larger community. Future projects will focus on adapting this strategy for dissemination to other urban jails, including those represented by our dissemination consultants, in HIV hotspots nationally to maximize its impact on the HIV epidemic for underserved minority communities.

## Abbreviations

CDC: Centers for Disease Control and Prevention
CL: criminal legal
DCJ: Dallas County Jail
EHE: Ending the HIV Epidemic
EHR: electronic health record
EPIS: exploration, preparation, implementation, sustainment
HARC: HIV/AIDS Re-Entry Coalition
HIPAA: Health Insurance Portability and Accountability Act
IRB: Institutional Review Board
MSM: men who have sex with men
PCCI: Parkland Center for Clinical Innovation
PrEP: pre-exposure prophylaxis
STI: sexually transmitted infection
